# Anticancer Properties of Natural Products

**DOI:** 10.1155/2015/242070

**Published:** 2015-05-20

**Authors:** István Zupkó, Walter Jaeger, Zeki Topcu, Chin-Chung Wu

**Affiliations:** ^1^Department of Pharmacodynamics and Biopharmacy, Faculty of Pharmacy, University of Szeged, Szeged 6720, Hungary; ^2^Department of Clinical Pharmacy and Diagnostics and Faculty Center of Pharmaceutical Sciences, University of Vienna, 1090 Vienna, Austria; ^3^Department of Pharmaceutical Biotechnology, Faculty of Pharmacy, Ege University, 35100 Izmir, Turkey; ^4^Graduate Institute of Natural Products, College of Pharmacy, Kaohsiung Medical University, Kaohsiung 80708, Taiwan

Although natural products have been used by man since ancient times, it was only in the past century that active components of medicinal plants became available in chemically pure form. An impressive number of natural products have been introduced into medical practice and also used as lead or model molecules for structure optimization and for the development of more potent or better-tolerated drugs. A recent comprehensive review found that only 20.2% of the anticancer agents approved in the period 1981–2010 were purely synthetic, whereas the remaining 79.8% were natural products or inspired by natural products (D. J. Newman and G. M. Cragg, “Natural products as sources of new drugs over the 30 years from 1981 to 2010” Journal of Natural Products, vol. 75, no. 3, pp. 311–335, 2012), indicating that structures from nature are indispensable in anticancer lead-finding research.

In this special issue, we present a set of original research papers concerning various aspects of the antiproliferative properties of plant extracts or isolated natural products. Three of them describe investigations with promising extracts. The methanolic root extract of* Sclerocarya birrea* gave rise to oxidative stress and apoptosis in HepG2 cells (M. F. Armentano et al.). Fractions of the methanolic extract of* Croton sphaerogynus* exhibited antiproliferative activity against adherent cancer cell lines (K. P. dos Santos et al.). The mechanism of the anticancer action of the ethyl acetate extract of the traditional Chinese medicinal plant* Euphorbia helioscopia* was explored through the use of a hepatocellular carcinoma xenograft model in nude mice. The treatment with the extract resulted in apoptosis induction, decreased tumor growth, and invasion (J. Cheng et al.). Two papers in this issue concentrate on the isolation and subsequent investigation of natural products. Various antiproliferative xanthones were isolated from* Garcinia* species and two of them directly inhibited JAK kinase (L. Xu et al.). The natural products hamigerone and radicinol, isolated from fungi by means of a bioassay-guided procedure, exhibited antiproliferative and proapoptotic properties in adherent cancer cell lines without affecting the viability of normal cells (P. Giridharan et al.). Two further publications deal with previously identified compounds that were earlier not investigated in detail oncopharmacologically. Vitamin K1 displays antiproliferative activity, causes cell cycle arrest, and induces apoptosis in human colon cancer cell lines (A. Orlando et al.). The sesterterpene heteronemin exerted considerable growth-inhibiting activity against human cancer cell lines, including A498 renal carcinoma cells, resulting in apoptosis through the mitochondrial pathway and also autophagy in A498 cells (S.-Y. Wu et al.). Two papers describe potentially relevant interactions between natural products and therapeutically utilized anticancer agents. Curcumin and one of its analogues exhibited additive properties with the proteasome inhibitor bortezomib against HL60 cells (L. I. Nagy et al.). Three ecdysteroids sensitized both multidrug-resistant and nonresistant adherent cell lines towards traditional anticancer agents, including doxorubicin (A. Martins et al.). One of the presented papers reports on the validation of an* in vitro* method for the screening of potential anticancer extracts or compounds. Instead of the use of cultured cancer cell lines, slices of breast cancer tissues obtained from surgery were incubated with antiproliferative agents and the viabilities of the slices were determined (I. E. Carranza-Torres et al.).

We strongly trust that these publications in this special issue will contribute to the development of natural product-based and innovative drug candidates.

